# Cardiovascular magnetic resonance assessment of abdominal adiposity predicts early sub-clinical atherosclerosis in young adults: importance of relative adiposity

**DOI:** 10.1186/1532-429X-11-S1-P64

**Published:** 2009-01-28

**Authors:** Ilias Kylintireas, Jonathan Diesch, Corinne Trevitt, Merzaka Lazdam, Colin Cunnington, Robin Choudhury, Stefan Neubauer, Paul Leeson

**Affiliations:** University Of Oxford, Oxford, UK

**Keywords:** Cardiovascular Magnetic Resonance, Visceral Adipose Tissue, Abdominal Adiposity, Plaque Index, Turbo Spin Echo

## Introduction

Gross obesity has a significant adverse impact on the cardiovascular system. The clinical importance of the normal variation in body fat seen in the general population during early adulthood is unknown. Cardiovascular magnetic resonance imaging offers the unique capability for clear depiction and accurate quantification of adipose tissue alongside an effective non invasive and radiation free assessment of both early and advanced atherosclerotic disease. We investigated the relation between abdominal adiposity and early sub-clinical carotid atherosclerosis.

## Purpose

We related CMR assessment of abdominal adiposity to sub-clinical carotid atherosclerosis in a young non-obese population, free of classical cardiovascular risk factors.

## Methods

**Figure 1:**
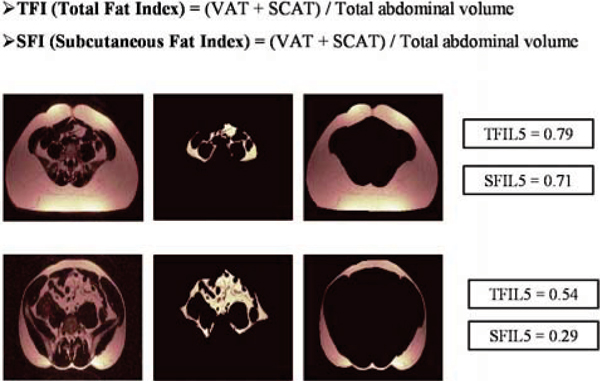
**Relative abdominal adiposity indices**.

Forty young volunteers (aged 23–33) (without any history of cardiovascular disease, gross obesity [mean BMI = 24.1 kg/m^2^ (± 4.3)] or classical cardiovascular risk factors) underwent CMR for abdominal adipose tissue quantification and carotid atheroma burden measurements. A Water Suppression (WS) T1 weighted (T1W) Turbo Spin Echo (TSE) multi-slice sequence was used for visceral adipose tissue (VAT) and abdominal subcutaneous adipose tissue (SCAT) measurement. We acquired 2 sets of 5 transverse slices (6 mm thick/1 mm gap), one positioned at the level of the fifth lumbar vertebrae (L5) and one at the level of the second lumbar (L2) vertebra. A semi-automated volumetric method was used to measure VAT and SCAT volumes for each set of slices (VATL2 and SCATL2 for the L2 level, and VATL5 and SCATL5 for the L5 level). To obtain an index of relative adiposity we derived a Total Fat Index (TFI) and Subcutaneous Fat Index (SFI) at each level. TFI was defined as the sum of VAT and SCAT volumes divided by overall abdominal volume and SFI as the SCAT volume divided by abdominal volume (Figure 1).

T1 weighted black blood turbo spin echo (TSE) cross-sectional images of both carotid arteries, centred at the lowest bifurcation were used for atheroma burden measurements (plaque index represented cross-sectional vessel wall area/total cross-sectional vascular area). Plaque index was averaged for the common carotid (CPI), the carotid bulb (BPI) and the internal carotid artery (IPI).

## Results

VAT and SCAT absolute measurements at both levels, TFIL2 and SFIL2 as well as body mass index (BMI), waist hip ratio (WHR), waist and hip circumferences, age and sex did not correlate with carotid atheroma burden. However, both TFIL5 and SFIL5 correlated with CPI (r = 0.359, P < 0.05 and r = 0.397, P < 0.05 respectively) and strongly correlated with BPI (r = 0.435, P < 0.05 and r = 0.428, P < 0.05 respectively).

Applying respective multiple regression analysis models (including applicable risk factors, demographics and anthropometric measurements) TFIL5 and SFIL5 emerged as sole independent predictors of atheroma burden both at the common carotid [β = 0.1905(± 0.09), P < 0.05, R^2^ = 0.20 and β = 0.1584(± 0.06), P < 0.05, R^2^ = 0.23 respectively] and the carotid bulb [β = 0.1862(± 0.09), P < 0.05, R^2^ = 0.19 and β = 0. 0.1294 (± 0.06), P < 0.05, R^2^ = 0.23 respectively].

## Conclusion

Relative increases in both total and subcutaneous abdominal adiposity across the normal ranges in a young adult population are associated with early sub-clinical atherosclerosis. Our observations illustrate the role of CMR in accurate evaluation of abdominal adiposity. Variation in abdominal fat relative to abdominal size may be of particular importance and abdominal adiposity at the fifth lumbar vertebral level may be the most relevant position to evaluate cardiovascular risk.

